# Miss Nott of St. Brenda's

**Published:** 1954

**Authors:** 


					MISS NOTT OF ST. BRENDA S
tv/ttoc mott OF ST. BRENDA S
Hth olber, ,?3. Mjss No?
aft ur0n of this Hospital. St. Brenda s began as a consul_
tamrstlflrSt Wor,d War- the movin8 Spi"tS lng,he .930s the obstetric side
deVf , turning to practice after the war years. the Nursing Home was
s .?Jed at the expense of the surgical and medica , an ^ Redland.
\vlwy faVoured by many of the general practit,oners m CM or^and
mS, n COmin8 of th<= National Health Service it was incorporate
roup as a General Practitioner Maternity ospita . exag-
LJWghout her twenty-five years Miss Nott has lived, m. ? her
and j? Say s^e *s esteemed by the doctors, e in congratulate
Wal07d by succeeding generations of mothers in Bristol We^ongr
n thank her on behalf of the profession for the wor s ic

				

